# Aging Phenotypes of Common Marmosets (*Callithrix jacchus*)

**DOI:** 10.1155/2012/567143

**Published:** 2012-03-04

**Authors:** Corinna N. Ross, Kenneth Davis, Georgina Dobek, Suzette D. Tardif

**Affiliations:** Department of Cellular and Structural Biology, Barshop Institute for Longevity and Aging Studies, University of Texas Health Science Center at San Antonio, 15355 Lambda Dr. STCBM Bldg, San Antonio, TX 78245, USA

## Abstract

Characterizing the phenotypic changes associated with aging in a short-lived primate is necessary in order to develop better translational models for human health, aging, and disease research. A population of conventionally housed marmoset monkeys was assessed to determine if phenotypes of body composition, hematology, and morphometrical measures were associated with age or risk of death. We found that the cause of mortality in older marmosets was more likely to be due to cardiac and chronic kidney disease than in younger marmosets. Older marmosets have decreased fat mass, morphometric measures, and serum albumin. Older marmosets are more likely to show a modified posture while at rest and this modified posture was significantly associated with an increased risk of imminent death. These assessments provide an initial definition of aged health in marmosets and a base for future translational aging research with this species.

## 1. Introduction

The relationships between health, aging, tissue function, and disease in primates and humans is often not well-modeled by rodent studies [1]. Nonhuman primates are our closest evolutionary relatives and as such are more similar to humans in terms of anatomy, embryology, fetal development, immunology, biochemistry, gene interactions, sensory apparatus, and overall physiological and psychological function than any other animal group. As a consequence, research with nonhuman primates is particularly relevant for the understanding of human health, disease, and therapeutics. The characterization of aging in a short-lived primate will open new possibilities for the assessment of health in the context of aging.

 Marmosets are small new world primates that offer a valuable resource as an animal model to examine adult disease risk, aging, and functional decline because they have the shortest average lifespan and fastest reproduction of any anthropoid primate [2]. Additionally, the long-standing use of marmosets as a model for family interactions, hormonal development, reproductive output, and medical research has resulted in a large base of average values for growth, body weight, and hematological measures. Marmosets are sexually monomorphic, and adults weigh an average of 300–500 grams in captivity [2]. They typically produce litters consisting of fraternal twins, with a gestational length of 143 days. Marmosets reach sexual maturity at approximately eighteen months, and the average lifespan in captivity for *Callithrix jacchus *is 4 to 6 years [2–4]. Marmosets are often considered “aged” at 8 years of age at which point studies have noted fibrous cartilaginous changes in intra-articular disks, *β*-amyloid deposition in the cerebral cortex, and reduced neurogenesis in the hippocampus [5–8]. The maximum lifespan reported for marmosets in captivity is 16 years; however, the population of animals aged 13–16 in any captive colony is very sparse, and the oldest animal in the Southwest National Primate Research Center (SNPRC) colony history was 13.7 [4]. The short lifespan of these primates along with the fast reproduction and recent improvements in husbandry results in the ability to form large populations of aged adults. Marmosets are also easily handled and do not carry many of the infectious zoonotic agents common to other primates currently used in biomedical research. Additionally, there is no evidence of density-dependent deaths for group-housed individuals related to increased aggression or competition as is commonly seen in many other primate species such as macaques and baboons. All of these factors contribute to making the marmoset an ideal model for the studies of biological aging and in particular the development of an animal model of functional decline and health span [9].

Studies of health and physical condition in humans have often focused on a core group of characters including body weight, body composition (fat and lean mass), activity patterns, inflammation, adiposity, gait, overall strength, muscle size, muscle strength, appetite, social function, and cognitive function [10, 11]. In order to examine functional decline associated with aging in marmosets we examined a broad spectrum of phenotypes relative to locomotion, body composition, and hematological markers. In particular we were interested in whether body composition is associated with age in marmosets. If marmosets exhibit functional decline with age that is similar to human decline then we predict significant losses in lean mass associated with age as is seen with sarcopenia for humans. Secondly, we were interested in whether morphometric measures, hematology, and behavioral activity were associated with age in marmosets. We predicted significant increases in inflammatory status, loss of muscle mass, and increased rates of resting and adjusted posture as a function of age. Lastly, we were interested in whether any of these biological markers were predictive of forthcoming death in marmosets.

## 2. Methods

### 2.1. Characterization of the Colony

The study population was chosen from the conventional breeding colony of *Callithrix jacchus* housed at the Southwest National Primate Research Center (SNPRC) in San Antonio, TX. All animal studies were reviewed and approved by the Institutional Animal Care and Use Committee at the Southwest Foundation for Biomedical Research, the host institution of SNPRC. The colony was established in 1994 by SDT with founding animals from a variety of established marmoset breeding colonies, none of the founding animals or animals imported into the colony at any time point were wildly caught. The SNPRC colony was a closed colony from 2000–2005 and no animals were imported from other colonies. The colony underwent significant growth during 2006–2010, with importation from a number of breeding colonies [9]. All animals in the colony are typically maintained on a diet which includes a purified gel-based diet (Teklad) [12]. Animals are group-housed as breeders and their offspring until juveniles become sexually mature (around two years of age) at which point they are removed from their natal group to become breeders themselves or are singly housed for experimental studies. An analysis of mortality patterns for breeding animals found that adult death was significantly reduced during the time the colony as closed (2000–2005) [9]. The median lifespan for animals surviving to at least the age of six months in this colony is 5.76 years and 6.48 for animals surviving to age of 2 [9]. Marmosets in this conventional colony are not specific pathogen free (SPF) and are not regularly screened for any infectious agents. Veterinary intervention is rare in the colony but most frequently animals are treated as necessary for symptoms of gastrointestinal disease with agents such as probiotics, and if they culture positively for *Giardia* they are treated with Tinidazole. Necropsy reports were analyzed to determine causes of mortality for deaths occurring between 2002 and 2011. Subjects were limited to those that had survived to at least six months of age and were not euthanized under an experimental or colony management protocol, *n* = 150; of these 99 were under 6 years of age and 51 were over 6 years.

### 2.2. Body Composition

Seventy-nine male marmosets ranging in age from young adulthood (2 years) to 13.7 years, the maximum lifespan in the SNPRC colony, and 39 nonpregnant females ranging in age from 2 to 10.7 years of age were examined for body composition (body mass, fat mass, and lean mass). In order to assess body composition animals were fasted overnight, weighed, and removed from the home cage via capture in a nest box. Body composition was analyzed using a qualitative magnetic resonance machine (EchoQMR) which has previously been validated for use in the marmoset to assess lean and fat mass [13]. The age class of the subjects were split into under 4 (young adult), 4–7 years of age (middle age adult), and over 8 years of age (older adult). An analysis of variance was used to evaluate the relationship between body composition measures, age, and gender of the subjects.

Additionally, for 46 animals in the colony multiple body composition measurements were made between 1 and 3 years following the first assessment. The average time between assessments was 2.3 years. For this data set there were 19 females ranging in age from 2.2 to 7.3 years of age and 27 males ranging in age from 2.6–8.3 years of age. For this data the change in age and the change in body mass variables were analyzed using correlations in SPSS.

### 2.3. Aging Phenotype

A subset of fifty males was further assessed for morphometrics, basic hematology, CRP concentration, and activity (scored during behavioral observations). For this data collection 2 mL of blood was gathered via the femoral vein from fasted animals. The blood was then sent to the SNPRC clinical pathology laboratory for basic hematology and CRP measurement. Morphological measures were taken using calipers including thigh-knee length and knee-heel length. A measuring tape was used to measure proximal thigh, medial thigh, distal thigh, proximal calf, medial calf, distal calf, and abdominal circumference. All measurements were made in triplicate and then averaged. Home cage behavior data was collected over two thirty-minute periods randomly scheduled over the course of the study using the observer software (Noldus). Males were observed using all occurrences methods and were scored as active (leaping, or quadrupedal motion) or inactive (sleeping, sitting, or stretching). Stretching was defined as a stationary adjusted posture in which the animal supports its weight with two limbs, typically the forelimbs, and the rest of the body is extended. The durations for all behaviors were averaged across observations. These data were then analyzed in SPSS to assess the correlations between the variables and the age, weight, fat, and lean mass of the subjects. In order to evaluate whether the variables measured were associated with a risk of death, the time from measurement to death for each individual was entered into a Cox regression model in SPSS. None of the subjects took part in other experimental or terminal protocols following the measures, and the data were censored at 2.7 years following the initial measure.

## 3. Results and Discussion

### 3.1. Characterization of the Colony

**Figure 1:**
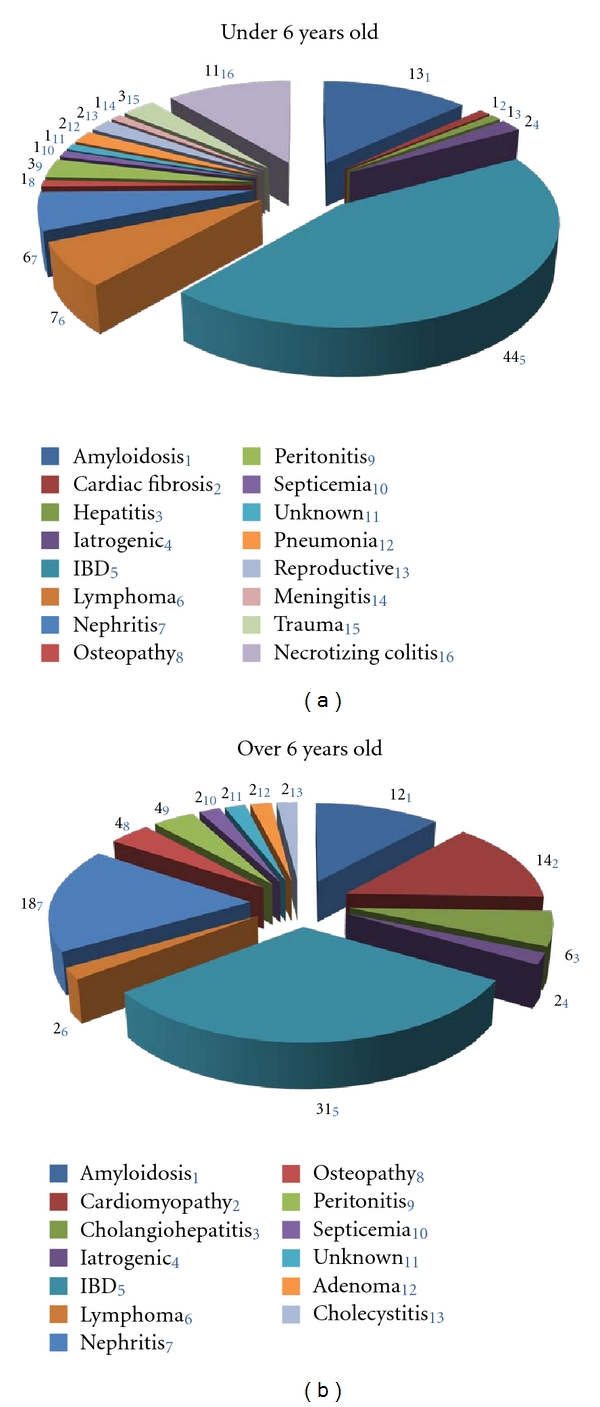
Causes of mortality for marmosets at SNPRC 2002–2011 under the age of 6 years (a), and over the age of 6 years (b). IBD: inflammatory bowel disease, iatrogenic: death from a complication associated with anesthesia.

The New England Primate Center reported that pathologies and causes of mortality differ by age for marmosets. In animals less than 6 years of age principal causes of death were trauma, inflammatory bowel disease, sepsis, and bacterial infection. In aged animals common causes of death included neoplasia, chronic renal disease, amyloidosis, and diabetes [9]. A previous report from the SNPRC marmoset colony noted predominant causes of death to be colitis, lymphosarcoma, amyloidosis, nephritis, and enteritis [14]. Analyzing the pathologies and causes of death noted for the SNPRC colony between 2002 and 2011 we find that the most noted causes of death for animals under the age of 6 are irritable bowel disease (enteritis, colitis), amyloidosis, and necrotizing colitis (Figure 1(a)). For animals over six years of age the most common causes of death in the SNPRC colony were irritable bowel disease, nephritis, cardiomyopathy, and amyloidosis (Figure 1(b)). The increased prevalence of cardiac and chronic kidney disease in the aged marmoset population may be particularly important for future modeling of cardiovascular and renal health in aging.

### 3.2. Body Composition

**Figure 2:**
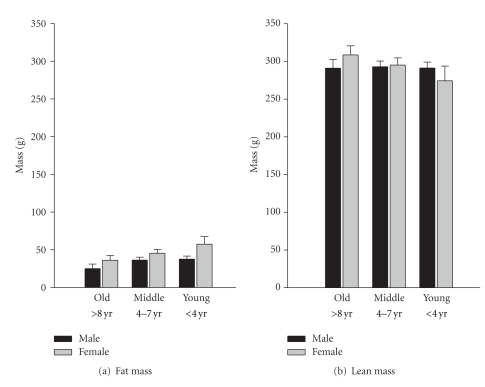
Mass varies in marmosets by both age and gender.

For male marmosets, age was positively associated with the absolute lean mass (*r* = 0.235, *P* = 0.037, *n* = 79), but was not associated with fat mass (*r* = −0.109, n.s.). For females, age was positively associated with absolute lean mass (*r* = 0.471, *P* = 0.002, *n* = 39) and was negatively associated with the absolute fat mass (*r* = −3.93, *P* = 0.013). Analysis of variance revealed that marmosets differ due to their gender for absolute fat (F(1,117) = 6.961, *P* = 0.01) and fat-lean mass ration (F(1,117) = 12.148, *P* = 0.00), but not weight. Differences also existed in association with the age class of the subjects for absolute fat mass (F(2,117) = 3.341, *P* = 0.039) and fat-lean mass ration (F(2,117) = 5.393, *P* = 0.006); all age classes differ from each other in posthoc analysis *P* < 0.05 (Figure 2). The subjects that were assessed multiple times using QMR were found to have a significant negative relationship between the change in age and the change in fat mass (*r* = −0.355, *P* = 0.016, *n* = 46), but there were no longitudinal effects on lean mass in these subjects.

### 3.3. Aging Phenotype

**Figure 3:**
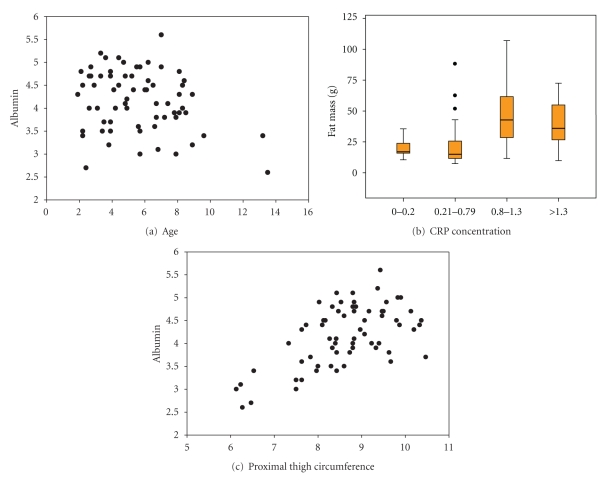
For male marmosets albumin concentrations are found to be negatively correlated with age (a), CRP is correlated with fat mass (b), and albumin is positively correlated with proximal thigh circumference (c).

While age and fat mass were not found to be significantly correlated for males, many human population studies find that morphometric and hematological values can be associated with both age and obesity status [10, 11, 15]. In order to examine the relationship between the variables measured and age partial correlations controlling either age or fat mass were done to examine the variables: proximal thigh circumference, abdomen circumference; albumin, red blood cell count, hemoglobin, hematocrit, and CRP concentrations; resting and stretching behaviors (Table 1). Of interest age was negatively related to albumin concentration (*r* = −0.323, *P* = 0.02) (Figure 3(a)). CRP was found to not be related to age, but was related to fat mass (*r* = 0.563, *P* = 0.00) with higher median fat mass being associated with higher CRP concentrations (Figure 3(b)). Average proximal thigh circumference was not surprisingly associated with measures of both body mass and age, but interestingly a relationship between thigh circumference and albumin concentrations remained even when controlled for both age and fat mass (*r* = 0.451, *P* = 0.007) (Figure 3(c)). The behavioral measure of an adjusted posture, stretching, was positively associated with age (*r* = 0.341, *P* = 0.013) and remained positively associated with CRP concentrations when age and fat mass were controlled (*r* = 0.622, *P* = 0.000). Impaired mobility, and low serum albumin concentrations are highly correlated with increased risk for sarcopenia, disability and morbidity in humans [16].

In order to determine whether any of the aging phenotype variables were predictive of death within 24 months following the study, data from 47 of the 50 animals that were originally assessed were entered into a Cox regression survival analysis. Three of the original animals were culled and were excluded from the hazards analysis. Twenty-five animals died naturally in the defined time frame, five died within 6 months of the study, 11 more had died within 12 months of the study, and a further 9 animals had died within 24 months following the study; the remaining 22 animals were censored for the analysis. The Cox regression survival analysis was done with the entry method, specifically looking at the variables defined above while controlling for age (Table 2). The only significant factor was the behavioral measure, stretch, suggesting that animals displaying this adjusted posture that may be indicative of pain or discomfort, have a higher risk of death. Interestingly, previous work with a mouse model of arthritis found that the posture of the mouse when stationary significantly predicted and predated the onset of arthritis and morbidity [17]. The derivation of biomarkers that are highly associated with the increased risk of death is extremely important for the development of models of health and future intervention testing. 

## 4. Conclusions

The ability to rapidly and easily measure a large number of hematological, morphological and body compositional variables in a small nonhuman primate makes the marmoset an ideal model for future studies of aging and health span. In this study we identified a number of markers that are associated not only with aging but also with risk of death. We found that aged marmosets were more likely to suffer from cardiac and renal failure than were younger marmosets. Rather than lean mass declining with age as would be predicted for sarcopenia-associated changes, in fact the marmosets' lean mass increases with age while fat mass is lost. Serum albumin was found to be significantly lower in aged marmosets than in young marmosets and was also associated with morphological measures of thigh circumference; this is analogous to results described for elderly humans. While CRP concentrations were not associated with age, they were positively associated with the modified posture noted as stretching, which was the only factor to significantly predict the risk of death when age was removed from the model. Perhaps animals displaying this adjusted posture are suffering from increased discomfort and inflammation. Frailty in humans is often associated with increased inflammatory status and decreased motor abilities; in fact one of the behavioral phenotypes used to evaluate frailty is the ability of the patient to rise from a seated position without modifying their posture [10, 11, 15]. 

 This paper was the first examination of phenotypes specifically to examine the relationship with age and health status in marmosets. This initial descriptive data is necessary for the further model development and initiation of aging research in marmosets. The results regarding body composition and hematological values and their relation to age closely resemble those previously reported in aging rodents and human populations. Analyses of the relationships among these variables provide operational definitions of “health” in this species that can be related back to human studies and provide a future bridge to examine preventatives, interventions or therapeutics. It will also provide a firm base to which additional phenotypic domains, such as cognitive function or cardiac function, can easily be added in the future.

## Figures and Tables

**Table 1:** Correlations for aging phenotype variables and age, or fat and lean mass (*partial correlation controlling for age) for 50 male marmosets.

Variable	Age	Fat*	Lean*
Proximal thigh	*r* = −0.311, *P* = 0.026	*r* = 0.598, *P* = 0.000	*r* = 0.773, *P* = 0.000
Abdominal circ	*r* = −0.075, n.s.	*r* = 0.308, *P* = 0.04	*r* = 0.398, *P* = 0.007
Albumin	*r* = −0.323, *P* = 0.022	*r* = 0.488, *P* = 0.003	*r* = 0.636, *P* = 0.000
Red blood cell	*r* = −0.18, n.s.	*r* = 0.424, *P* = 0.004	*r* = 0.399, *P* = 0.007
Hemoglobin	*r* = −0.24, n.s.	*r* = 0.386, *P* = 0.022	*r* = 0.424, *P* = 0.011
Hematocrit	*r* = −0.181, n.s.	*r* = 0.371, *P* = 0.028	*r* = 0.458, *P* = 0.006
CRP	*r* = 0.101, n.s.	*r* = 0.562, *P* = 0.000	*r* = 0.515, *P* = 0.002
Behavior: Rest	*r* = 0.036, n.s.	*r* = 0.064, n.s.	*r* = −0.033, n.s.
Behavior: Stretch	*r* = 0.341, *P* = 0.013	*r* = 0.119, n.s.	*r* = 0.265, n.s.

**Table 2:** Cox regression survival analysis for aging phenotypes in marmosets, *all variables added to the model by entry controlling for age.

Variable	B	Sig (*P*)	Hazard
Age	0.293	0.000	1.341
Proximal thigh*	−0.252	n.s.	0.777
Albumin*	−0.794	n.s.	0.452
Red blood cell*	0.248	n.s.	1.282
Hemoglobin*	−0.461	n.s.	0.63
Hematocrit*	0.117	n.s.	1.125
Fat*	−0.002	n.s.	0.998
Lean*	−0.015	n.s.	0.985
Behavior-Stretch*	0.208	0.012	1.231

## References

[1] Shi Y, Buffenstein R, Pulliam DA, Van Remmen H (2010). Comparative studies of oxidative stress and mitochondrial function in aging. *Integrative and Comparative Biology*.

[2] Tardif SD, Smucny DA, Abbott DH, Mansfield K, Schultz-Darken N, Yamamoto ME (2003). Reproduction in captive common marmosets (*Callithrix jacchus*). *Comparative Medicine*.

[3] Ross CN, Fite JE, Jensen H, French JA (2007). Demographic review of a captive colony of callitrichids (*Callithrix kuhlii*). *American Journal of Primatology*.

[4] Smucny DA, Abbott DH, Mansfield KG (2004). Reproductive output, maternal age, and survivorship in captive common marmoset females (*Callithrix jacchus*). *American Journal of Primatology*.

[5] Abbott DH, Barnett DK, Colman RJ, Yamamoto ME, Schultz-Darken NJ (2003). Aspects of common marmoset basic biology and life history important for biomedical research. *Comparative Medicine*.

[6] Berkovitz BKB, Pacy J (2000). Age changes in the cells of the intra-articular disc of the temporomandibular joints of rats and marmosets. *Archives of Oral Biology*.

[7] Geula C, Nagykery N, Wu CK (2002). Amyloid-*β* deposits in the cerebral cortex of the aged common marmoset (*Callithrix jacchus*): incidence and chemical composition. *Acta Neuropathologica*.

[8] Leuner B, Kozorovitskiy Y, Gross CG, Gould E (2007). Diminished adult neurogenesis in the marmoset brain precedes old age. *Proceedings of the National Academy of Sciences of the United States of America*.

[9] Tardif SD, Mansfield KG, Ratnam R, Ross CN, Ziegler TE (2011). The marmoset as a model of aging and age-related diseases. *Institute for Laboratory Animal Research Journal*.

[10] Fried LP, Ferrucci L, Darer J, Williamson JD, Anderson G (2004). Untangling the concepts of disability, frailty, and comorbidity: implications for improved targeting and care. *Journals of Gerontology*.

[11] Walston J, Hadley EC, Ferrucci L (2006). Research agenda for frailty in older adults: toward a better understanding of physiology and etiology: summary from the American Geriatrics Society/National Institute on Aging research conference on frailty in older adults. *Journal of the American Geriatrics Society*.

[12] Tardif S, Jaquish C, Layne D (1998). Growth variation in common marmoset monkeys (*Callithrixjacchus*) fed a purified diet: relation to care-giving and weaning behaviors. *Laboratory Animal Science*.

[13] Tardif SD, Power ML, Ross CN, Rutherford JN, Layne-Colon DG, Paulik MA (2009). Characterization of obese phenotypes in a small nonhuman primate, the common marmoset (*Callithrix jacchus*). *Obesity*.

[14] David JM, Dick EJ, Hubbard GB (2009). Spontaneous pathology of the common marmoset (*Callithrix jacchus*) and tamarins (*Saguinus oedipus, Saguinus mystax*). *Journal of Medical Primatology*.

[15] Mehta KM, Pierluissi E, Boscardin WJ (2011). A clinical index to stratify hospitalized older adults according to risk for new-onset disability. *Journal of the American Geriatrics Society*.

[16] Visser M, Kritchevsky SB, Newman AB (2005). Lower serum albumin concentration and change in muscle mass: the health, aging and body composition study. *American Journal of Clinical Nutrition*.

[17] Williams JM, Zurawski J, Mikecz K, Glant TT (1993). Functional assessment of joint use in experimental inflammatory murine arthritis. *Journal of Orthopaedic Research*.

